# Observational Study of the Association Between Oral *Helicobacter pylori*, Fixed Orthodontic Appliances, and Gastric Cancer Risk

**DOI:** 10.3390/jcm15072785

**Published:** 2026-04-07

**Authors:** Ioana Maria Crișan, Alex Crețu, Sorana-Maria Bucur

**Affiliations:** 1 Faculty of Medicine, George Emil Palade University of Medicine, Pharmacy, Science, and Technology of Târgu Mureș, 38 Ghe. Marinescu Street, 540142 Târgu Mureș, Romania; ioanamariacrisan16@gmail.com; 2 Department of Dentistry, Faculty of Medicine, Dimitrie Cantemir University of Târgu Mureș, 540545 Târgu Mureș, Romania; bucursoranamaria@gmail.com

**Keywords:** *Helicobacter pylori*, gastric cancer, oral microbiome, orthodontic appliances, oral reservoir, PCR detection, observational study

## Abstract

**Background:** *Helicobacter pylori* is a well-established risk factor for gastric carcinogenesis. Increasing evidence suggests that the oral cavity may serve as an extragastric reservoir for the bacterium, potentially contributing to persistent infection and reinfection. Orthodontic appliances can modify oral biofilm ecology and may facilitate bacterial colonization. This study aimed to investigate the association between oral *H. pylori* colonization and gastric cancer, while exploring the potential modifying role of fixed orthodontic appliances. **Materials and Methods:** In this cross-sectional observational study, 212 participants were recruited from gastroenterology and dental clinics between January 2023 and March 2025. Oral samples were collected and analyzed for *H. pylori* DNA using polymerase chain reaction (PCR). Gastric diagnoses were established through endoscopic examination and histopathological evaluation, classifying participants into gastric cancer, precancerous gastric lesions, non-atrophic gastritis, and control groups. Demographic, clinical, and oral health variables were recorded. Multivariable logistic regression models were used to evaluate the association between oral *H. pylori* detection and gastric cancer while adjusting for potential confounders, including age, sex, smoking status, oral hygiene indicators, and socioeconomic factors. **Results:** Oral *Helicobacter pylori* DNA was detected in 35/54 (64.8%) patients with gastric cancer, 30/56 (53.6%) with precancerous lesions, 21/52 (40.4%) with non-atrophic gastritis, and 15/50 (30.0%) controls. Gastric *H. pylori* infection was identified in 41/54 (75.9%) gastric cancer cases compared with 18/50 (36.0%) controls. Oral *H. pylori* positivity was more frequent among patients undergoing active orthodontic treatment (22/36, 61.1%) than among those without orthodontic appliances (79/188, 42.0%). In multivariable analysis, oral *H. pylori* positivity remained independently associated with gastric cancer (adjusted OR 3.02, 95% CI 1.51–6.03, *p* = 0.002). **Conclusions:** Our findings support an association between oral–gastric microbial interactions and *H. pylori*–associated disease, and suggest that the oral cavity may serve as a potential reservoir for gastric infection dynamics. The presence of orthodontic appliances may be associated with altered oral microbial ecology and could be linked to sustained *H. pylori* colonization.

## 1. Introduction

Gastric cancer remains a major global health burden, ranking among the leading causes of cancer-related mortality worldwide [1]. Despite a gradual decline in incidence in certain regions, largely attributable to improvements in sanitation, food preservation, and early detection strategies, it continues to exhibit marked geographic heterogeneity, with the highest prevalence observed in East Asia, Eastern Europe, and parts of Latin America [2,3]. Among the numerous environmental, dietary, and host-related factors implicated in gastric carcinogenesis, *Helicobacter pylori* infection represents the most consistently and causally associated risk factor, accounting for the majority of non-cardia gastric cancers [4,5].

*Helicobacter pylori* is a Gram-negative, microaerophilic bacterium that chronically colonizes the gastric mucosa and infects nearly half of the global population. In 1994, the World Health Organization classified *H. pylori* as a class I carcinogen, marking the first formal recognition of a bacterium as a direct cause of human cancer [6]. Extensive epidemiological, experimental, and clinical evidence has since established its central role in gastric tumorigenesis and revealed substantial interindividual variability in disease outcomes [6,7]. Although most infected individuals remain asymptomatic [8], a subset progresses along a well-characterized pathological continuum—from chronic active gastritis to atrophic gastritis, intestinal metaplasia, dysplasia, and ultimately adenocarcinoma—commonly referred to as the Correa cascade [9].

The oncogenic potential of *H. pylori* arises from a complex interplay between bacterial virulence factors, host genetic susceptibility, immune responses, and environmental modifiers. Among bacterial determinants, the cytotoxin-associated gene A (*cagA*) and vacuolating cytotoxin A (*vacA*) are the most extensively studied. *CagA*-positive strains are associated with increased gastric inflammation, epithelial disruption, and a significantly elevated risk of gastric cancer [10]. Following translocation into gastric epithelial cells via a type IV secretion system, *cagA* perturbs multiple intracellular signaling pathways, including SHP-2, MAPK, β-catenin, and NF-κB, thereby promoting aberrant cell proliferation, loss of polarity, genomic instability, and resistance to apoptosis [11]. Polymorphic variants of *vacA* further modulate epithelial injury, immune evasion, and mitochondrial dysfunction, amplifying carcinogenic signaling [11,12,13].

Chronic *H. pylori* infection induces persistent gastric inflammation characterized by immune cell infiltration and sustained production of pro-inflammatory cytokines and reactive oxygen species, promoting DNA damage, epigenetic alterations, and progressive glandular loss [14]. Host genetic polymorphisms affecting cytokine expression, including interleukin-1β, tumor necrosis factor-α, and interleukin-10, further modulate susceptibility to severe gastritis and malignant transformation, underscoring the importance of host–pathogen interactions in determining clinical outcomes [15].

Beyond the gastric niche, increasing evidence indicates that *H. pylori* may colonize extragastric reservoirs, particularly the oral cavity, where it can persist within dental plaque, saliva, and oral biofilms. The oral cavity has therefore been proposed as a potential reservoir facilitating transmission, gastric reinfection following eradication therapy, and long-term persistence of virulent strains [16]. Oral colonization may indirectly contribute to gastric carcinogenesis by influencing microbial translocation, modifying the oral–gastric microbiome axis, and sustaining low-grade inflammatory signaling [17]. Although its precise role remains incompletely defined, the frequent coexistence of oral and gastric *H. pylori* infection, along with the genetic similarity observed in some patients, suggests biological relevance that warrants further investigation [18,19]. However, whether oral colonization merely serves as a passive reservoir or actively contributes to gastric carcinogenesis remains poorly understood. Furthermore, the potential influence of dental interventions that alter oral microbial ecology, such as fixed orthodontic appliances, has rarely been examined in the context of *H. pylori* persistence and gastric cancer risk.

The recognition that gastric carcinogenesis is shaped not only by *H. pylori* infection itself but also by its interaction with microbial ecology, host immunity, and environmental exposures has reshaped current paradigms in prevention and management. While large-scale studies have demonstrated that *H. pylori* eradication reduces gastric cancer incidence, particularly when implemented before irreversible precancerous changes, eradication success varies according to bacterial resistance patterns, host factors, and the presence of potential reservoirs [20].

In this context, the oral microbiome has emerged as a dynamic and clinically relevant modifier of microbial persistence. Individuals undergoing orthodontic treatment, particularly those wearing fixed appliances, represent a distinct oral ecological niche characterized by increased plaque retention and impaired mechanical biofilm removal. Brackets, archwires, and ligatures create retentive surfaces that favor microbial maturation and increased microbial diversity, even in patients with adequate oral hygiene practices. Clinical studies have shown that fixed orthodontic appliances are associated with shifts toward more complex and potentially pathogenic oral biofilms [21,22]. With the global prevalence of orthodontic treatment increasing substantially over the past two decades, understanding its potential impact on microbial reservoirs relevant to systemic disease has become increasingly important.

**Figure 1:**
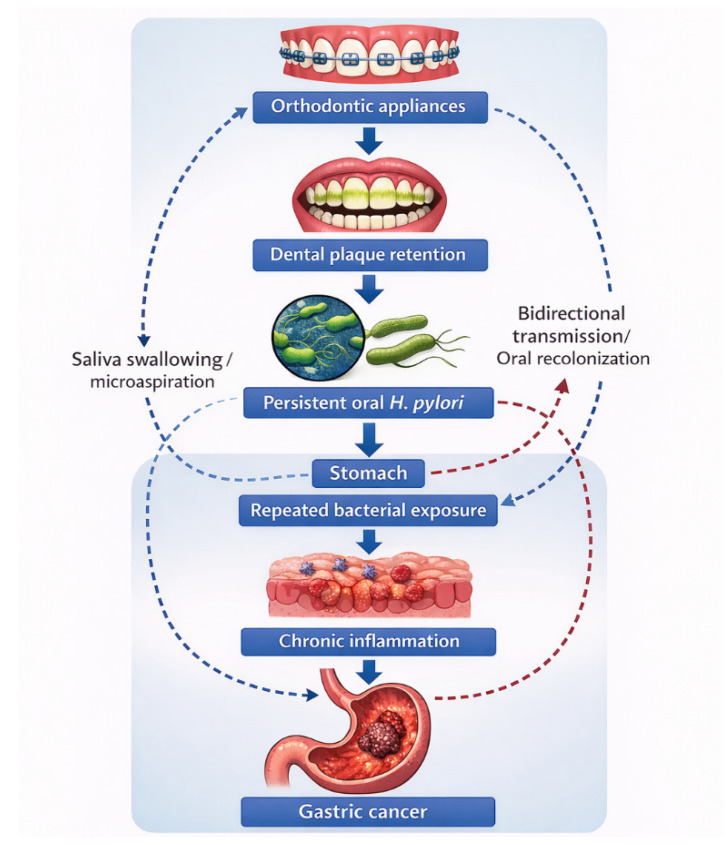
Proposed model of the oral–gastric *Helicobacter pylori* axis in gastric carcinogenesis.

Given the demonstrated ability of *H. pylori* to survive within dental plaque and microaerophilic oral biofilms, orthodontic treatment may inadvertently facilitate the persistence of oral *H. pylori* reservoirs [23]. This introduces orthodontic therapy as a potential modulator of the oral–gastric microbial axis, with implications for *H. pylori* infection dynamics and gastric carcinogenesis (Figure 1).

Fixed orthodontic appliances promote dental plaque retention and biofilm maturation, creating microaerophilic niches that may facilitate persistent oral colonization by *Helicobacter pylori*. Bacteria originating from oral reservoirs may repeatedly reach the gastric mucosa through saliva swallowing and may be associated with chronic inflammation and carcinogenic progression. Bidirectional transmission between oral and gastric niches may further sustain long-term bacterial persistence.

Against this background, the present study aimed to investigate the association between oral *Helicobacter pylori* colonization and gastric cancer and its precursor lesions, while exploring the potential modifying role of fixed orthodontic treatment. We hypothesized that orthodontic appliances may promote persistent oral *H. pylori* colonization and thereby influence the oral–gastric microbial axis involved in gastric carcinogenesis.

## 2. Materials and Methods

### 2.1. Study Design and Ethical Considerations

This study was conducted as an observational, cross-sectional clinical and molecular investigation to evaluate the association between *Helicobacter pylori* colonization of the oral cavity and gastric pathology, with particular emphasis on gastric cancer and its precursor lesions. The research was carried out through an interdisciplinary collaboration between the Faculty of Medicine of “Dimitrie Cantemir” University of Târgu Mureș, Romania, and the Department of Gastroenterology of “George Emil Palade”, integrating dental, microbiological, histopathological, and oncological expertise. Particular attention was given to oral conditions that modify microbial ecology, including the presence of fixed orthodontic appliances. Participants were recruited between January 2023 and March 2025. The study was conducted in accordance with the principles of the Declaration of Helsinki and approved by the Ethics Committee of the George Emil Palade University of Medicine, Pharmacy, Science, and Technology of Târgu Mureș. Ethical approval was granted under approval number 1970/15.12.2022.

All participants received detailed information regarding the study objectives, procedures, potential risks, and benefits, and provided written informed consent before enrollment. For patients undergoing upper gastrointestinal endoscopy, informed consent included permission for gastric sampling and the use of anonymized clinical and histopathological data for research purposes.

### 2.2. Sample Size Considerations

A minimum sample size of 200 participants was estimated to provide 80% statistical power to detect an odds ratio of at least 2.0 for the association between oral *H. pylori* detection and gastric cancer at a significance level of 0.05.

### 2.3. Study Population and Participant Selection

Participants were recruited consecutively from gastroenterology and dental outpatient clinics over a defined study period. The study population consisted of adult patients (≥18 years) undergoing upper gastrointestinal endoscopy for diagnostic or surveillance indications, as well as age- and sex-matched controls without known malignant disease.

Based on histopathological findings, participants were stratified into four groups: patients with histologically confirmed gastric adenocarcinoma; patients with precancerous gastric lesions, including chronic atrophic gastritis and/or intestinal metaplasia; patients with non-atrophic gastritis; and controls with normal gastric mucosa.

Exclusion criteria included prior *H. pylori* eradication therapy within six months preceding enrollment; use of antibiotics, proton pump inhibitors, or bismuth compounds within four weeks before sampling; previous gastric surgery; autoimmune gastritis; severe systemic disease; pregnancy; and ongoing oncological treatment such as chemotherapy or radiotherapy. To minimize transient alterations of the oral microbiome, patients undergoing active periodontal therapy or recent dental procedures within the previous four weeks were excluded.

Information regarding current or previous fixed orthodontic treatment was systematically recorded. Fixed orthodontic treatment was defined as the presence of bonded orthodontic appliances for at least 3 months. Data regarding the total duration of orthodontic treatment before enrollment were not systematically available and therefore were not included in the present analysis. Patients undergoing active orthodontic treatment were analyzed as a predefined subgroup.

All orthodontic appliances consisted of fixed stainless-steel bracket systems with similar archwire materials, and there was no substantial variation in appliance material composition. The specific bracket design (e.g., conventional versus self-ligating) was not systematically differentiated and was therefore not analyzed separately

### 2.4. Clinical and Oral Examination

All participants underwent a standardized oral examination performed by a calibrated general dentist. Oral hygiene status, periodontal condition, and the presence of active carious lesions were assessed using validated clinical indices. Plaque accumulation and gingival inflammation were recorded to account for their potential influence on oral microbial colonization. Plaque accumulation was assessed using the Silness–Löe Plaque Index, while gingival inflammation was evaluated using the Gingival Index.

The presence of fixed orthodontic appliances was documented during the examination, and plaque accumulation adjacent to orthodontic brackets was specifically noted. Demographic data and lifestyle factors, including oral hygiene habits, smoking status, alcohol consumption, dietary patterns, and socioeconomic background, were collected using a structured questionnaire, given their established association with both *H. pylori* prevalence and gastric cancer risk.

### 2.5. Sample Collection

#### 2.5.1. Oral Samples

Oral samples were collected before endoscopic procedures to prevent cross-contamination. Unstimulated saliva and supragingival dental plaque were obtained under standardized conditions using sterile instruments. Dental plaque was collected from multiple sites, including molar and premolar regions. Sterile sampling instruments were used for each site, and negative environmental controls were included to minimize contamination during collection and DNA extraction. In patients with fixed orthodontic appliances, plaque samples were preferentially collected from bracket-adjacent tooth surfaces, which represent areas of increased biofilm retention.

All samples were immediately placed in sterile tubes containing transport medium and stored at −80 °C until further processing.

#### 2.5.2. Gastric Samples

During upper gastrointestinal endoscopy, gastric biopsy specimens were obtained from the antrum and corpus in accordance with standard clinical guidelines. One biopsy specimen was used for rapid urease testing, while additional samples were fixed in formalin for histopathological examination or stored for molecular analyses when applicable.

#### 2.5.3. Detection of *Helicobacter pylori*

Gastric *H. pylori* infection status was determined using a combination of diagnostic methods, including histological examination, rapid urease testing, and molecular detection when available, in accordance with international consensus recommendations. Histological assessment was performed using hematoxylin–eosin and modified Giemsa staining.

#### 2.5.4. Oral Detection

Genomic DNA was extracted from oral samples using commercially available extraction kits following the manufacturer’s protocols. Detection of *H. pylori* DNA was performed by polymerase chain reaction (PCR), targeting conserved bacterial genes such as *16S rRNA*, *ureA*, or *glmM*, selected for their sensitivity and specificity in oral samples.

To explore virulence-associated pathogenicity, additional PCR assays were conducted to detect *cagA* and *vacA* genotypes when sufficient DNA quantity and quality were available. Appropriate positive and negative controls were included in all PCR assays.

PCR amplification was performed using specific primers targeting the *Helicobacter pylori 16S rRNA*, *ureA*, and *glmM* genes. Primer sequences and expected amplicon sizes are provided in Table 1. Amplification conditions consisted of an initial denaturation at 95 °C for 5 min, followed by 35 cycles of denaturation at 95 °C for 30 s, annealing at 55–60 °C for 30 s, and extension at 72 °C for 45 s, with a final extension at 72 °C for 7 min.

PCR amplification targeted the *Helicobacter pylori 16S rRNA*, *ureA*, and *glmM* (*ureC*) genes using previously validated primer sets widely used for molecular detection of *H. pylori*. Expected amplicon sizes correspond to published reference protocols.

Samples were considered positive for *Helicobacter pylori* when amplification of at least one of the targeted genes (*16S rRNA*, *ureA*, or *glmM*) was detected in the presence of appropriate positive and negative controls. This criterion was selected to maximize sensitivity for oral detection.

#### 2.5.5. Histopathological Evaluation

Gastric biopsy specimens were independently evaluated by experienced gastrointestinal pathologists who were blinded to oral microbiological findings. Gastritis severity, inflammatory activity, glandular atrophy, and intestinal metaplasia were graded according to the Updated Sydney System. Gastric cancer cases were further classified based on histological subtype and degree of differentiation.

### 2.6. Statistical Analysis

Statistical analyses were performed using dedicated statistical software. Descriptive statistics were used to summarize demographic, clinical, oral, and microbiological variables. Continuous variables were analyzed using Student’s *t*-test or nonparametric equivalents, while categorical variables were compared using chi-square or Fisher’s exact test, as appropriate.

Multivariable logistic regression models were constructed to assess the association between oral *H. pylori* detection and gastric cancer or precancerous lesions, adjusting for potential confounders including age, sex, smoking status, oral hygiene indices, socioeconomic factors, and the presence of fixed orthodontic appliances. Odds ratios (ORs) with 95% confidence intervals (CIs) were calculated, and a two-sided *p* value < 0.05 was considered statistically significant.

Missing data were minimal (<3% of observations) and were handled using complete-case analysis without imputation.

Analyses were performed using SPSS version 20 (IBM Corp., Armonk, NY, USA). Multicollinearity among independent variables was assessed using variance inflation factors (VIFs).

## 3. Results

### 3.1. Study Population Flow and Data Completeness

A total of 236 individuals were initially screened for eligibility during the study period (January 2023–March 2025). Twenty-four individuals were excluded before analysis due to predefined exclusion criteria, including recent antibiotic or proton pump inhibitor use (*n* = 11), prior Helicobacter pylori eradication therapy within six months (*n* = 7), recent dental procedures that affect the oral microbiota (*n* = 4), or incomplete clinical data (*n* = 2). The remaining 212 participants fulfilled all eligibility criteria and were included in the final analysis.

According to histopathological findings, participants were categorized into four diagnostic groups: gastric cancer (*n* = 54), precancerous gastric lesions (*n* = 56), non-atrophic gastritis (*n* = 52), and controls with normal gastric mucosa (*n* = 50).

All included participants underwent oral sampling and clinical examination. Gastric biopsy samples suitable for histopathological evaluation were available for all patients undergoing endoscopy. Molecular detection of *Helicobacter pylori* from oral samples was successfully performed in all cases. Occasional missing values for selected questionnaire variables (e.g., socioeconomic indicators) were minimal (<3%) and were handled by complete-case analysis in multivariable models.

**Figure 2:**
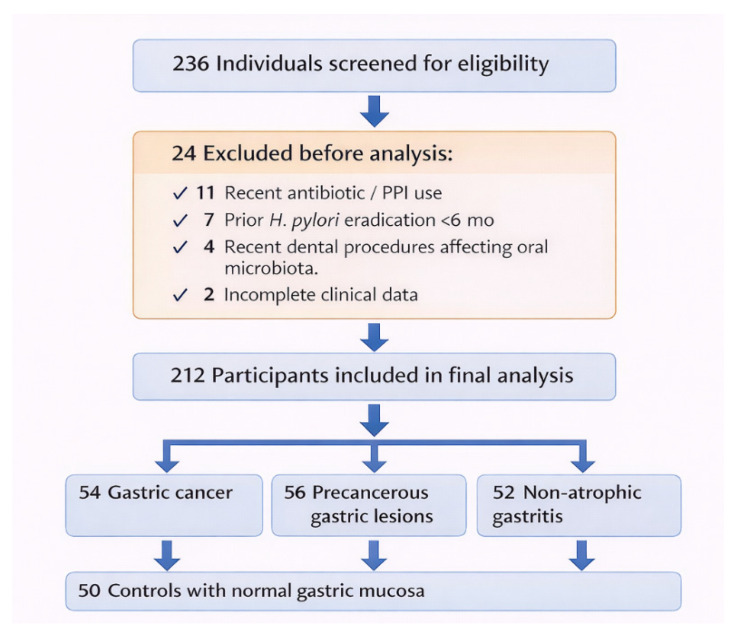
Study Population Flow Diagram illustrating participant screening, exclusion criteria, and final allocation into diagnostic groups included in the observational analysis.

The participant selection process is summarized in a study flow diagram (Figure 2).

### 3.2. Study Population Characteristics

A total of 212 participants were included in the final analysis after application of inclusion and exclusion criteria. Participants were distributed across the four predefined diagnostic groups: gastric cancer, precancerous gastric lesions, non-atrophic gastritis, and controls with normal gastric mucosa.

Patients diagnosed with gastric cancer were older compared with controls and exhibited a higher prevalence of established risk factors, including smoking. Fixed orthodontic treatment, either current or in the past, was documented in 58 participants (27.4%), with 36 patients (17.0%) undergoing active orthodontic therapy at the time of enrollment. Orthodontic treatment was more frequently observed among younger participants, but was present across all diagnostic categories.

Gastric *H. pylori* infection was detected significantly more frequently in patients with gastric cancer and precancerous gastric lesions compared to controls (Table 2).

Infection prevalence followed a clear gradient corresponding to disease severity, with the highest rates observed in gastric cancer patients.

The prevalence of gastric *H. pylori* infection increased progressively across disease categories, demonstrating progressively higher infection prevalence with increasing disease severity (Table 3). Differences across groups were assessed using the χ^2^ test.

### 3.3. Oral H. pylori Detection

Oral *H. pylori* DNA was detected in saliva and/or dental plaque samples across all groups. However, oral colonization rates increased progressively from controls to non-atrophic gastritis, precancerous lesions, and gastric cancer (Table 4). Patients with gastric *H. pylori* infection were significantly more likely to present oral *H. pylori* positivity.

All orthodontic appliances consisted of fixed stainless-steel bracket systems, ensuring comparable material composition across treated participants.

### 3.4. Fixed Orthodontic Treatment and Oral H. pylori

Patients undergoing active fixed orthodontic treatment exhibited significantly higher rates of oral *H. pylori* detection compared with patients without orthodontic appliances (Table 5). Among orthodontic patients, the highest positivity rates were observed in dental plaque samples collected adjacent to orthodontic brackets.

Patients undergoing active orthodontic treatment were significantly younger than those without appliances; therefore, age-adjusted regression models and interaction analyses were performed to account for potential confounding.

Interaction analysis indicated that the combined presence of oral *H. pylori* and orthodontic treatment was associated with higher odds of gastric cancer than either factor alone.

### 3.5. Virulence-Associated Genotypes in Oral Samples

Virulence-associated *H. pylori* genes were detected more frequently in oral samples from patients with gastric cancer (Table 6). The *cagA* gene was identified in a substantial proportion of oral *H. pylori*-positive gastric cancer patients, whereas its prevalence was lower among controls.

### 3.6. Multivariable Analysis

In multivariable logistic regression models adjusting for age, sex, smoking status, oral hygiene indices, socioeconomic factors, and gastric *H. pylori* infection, oral *H. pylori* positivity remained independently associated with gastric cancer. Fixed orthodontic treatment was independently associated with oral *H. pylori* detection and appeared to amplify the association between oral colonization and gastric cancer when both factors were present (Table 7). Model fit was assessed using the Hosmer–Lemeshow goodness-of-fit test.

Overall, these results demonstrate a strong association between oral *H. pylori* colonization and gastric cancer, with fixed orthodontic treatment emerging as a significant modifier of oral bacterial persistence. The combined presence of oral *H. pylori* and orthodontic appliances was associated with the highest odds of gastric cancer, supporting a possible association between oral ecological factors and gastric carcinogenesis.

## 4. Discussion

In this interdisciplinary clinical and molecular study, we identified a significant association between oral *Helicobacter pylori* colonization and gastric cancer, independent of several established risk factors. In addition, our findings suggest that fixed orthodontic appliances may act as ecological modifiers of the oral microbiome and may be associated with persistent oral reservoirs of H. pylori. Taken together, these observations support an association, suggesting that gastric carcinogenesis may be related not only to gastric infection itself but also to microbial dynamics occurring along the oral–gastric axis. This perspective expands the traditional stomach-centered model of *H. pylori* pathogenesis and highlights the oral cavity as a potentially relevant microbial niche in the natural history of gastric cancer. These findings highlight the importance of integrating oral health evaluation into clinical strategies to manage H. pylori infection and reduce gastric cancer risk [23,24].

The progressive increase in oral *H. pylori* detection observed across the gastric disease spectrum, from controls to non-atrophic gastritis, precancerous lesions, and gastric cancer, suggests that oral colonization may be associated with cumulative exposure to carcinogenic bacterial stimuli. This gradient is consistent with models of microbial persistence in which biofilm-associated bacterial reservoirs contribute to sustained host exposure and chronic inflammatory signaling. Previous studies have proposed the oral cavity as a potential extragastric reservoir for *H. pylori*, capable of harboring viable bacteria within dental plaque and microaerophilic niches [16]. Our findings extend these observations by demonstrating that oral colonization correlates with the severity of gastric pathology [23,24]. Although the cross-sectional design precludes causal inference, the strength and consistency of the association reinforce the biological plausibility of a bidirectional oral–gastric microbial relationship [25]. Because orthodontic treatment is more frequently performed in younger individuals, age-adjusted regression models were used to minimize potential confounding.

Interestingly, oral *H. pylori* positivity was observed in a proportion of control participants exceeding the prevalence of gastric infection. This finding may reflect the ability of *H. pylori* DNA to persist within oral biofilms independently of active gastric colonization. The oral cavity may harbor transient or low-level colonization, potentially representing prior exposure, environmental contamination, or nonviable bacterial DNA detected by PCR-based methods [19,23]. In addition, oral biofilms provide protective microenvironments that may support bacterial persistence even in the absence of detectable gastric infection. These findings are consistent with previous reports suggesting that oral detection of *H. pylori* does not always correlate with active gastric colonization and may represent an independent microbial reservoir [23,26].

Our study further highlights the potential relevance of *H. pylori* virulence determinants detected in oral samples. The higher prevalence of *cagA*-positive strains among patients with gastric cancer suggests that virulent genotypes may persist beyond the gastric mucosa and establish stable colonization within oral biofilms. The *cagA* oncoprotein is known to dysregulate multiple intracellular signaling pathways involved in epithelial proliferation, cytoskeletal organization, and inflammatory responses, including SHP-2, MAPK, β-catenin, and NF-κB signaling cascades [11,27]. Persistent exposure to virulence-associated strains could be associated with repeated gastric exposure to carcinogenic bacterial factors. The detection of these virulence-associated genotypes within the oral cavity raises the possibility that oral reservoirs may repeatedly reseed the gastric mucosa or sustain systemic inflammatory stimulation. Such mechanisms could contribute to recurrent infection or incomplete bacterial eradication observed in some patients. As participants were recruited from clinical settings where endoscopy was indicated, selection bias toward symptomatic individuals cannot be excluded.

A particularly novel aspect of this study is the identification of fixed orthodontic appliances as a potential ecological modifier of oral *H. pylori* colonization. To our knowledge, this is among the first clinical studies investigating orthodontic treatment as a potential ecological modifier of oral *H. pylori* persistence and its association with gastric carcinogenesis. Fixed orthodontic treatment creates retentive surfaces that favor plaque accumulation, biofilm maturation, and the formation of localized microaerophilic microenvironments within dental plaque [28]. These conditions may be particularly favorable for *H. pylori*, a microaerophilic organism that can survive within structured biofilm communities. Biofilm matrices provide physical protection from environmental stressors and antimicrobial agents while facilitating metabolic cooperation among microbial species. In this ecological context, orthodontic appliances may generate niches that could be associated with the persistence of *H. pylori* within the oral cavity. Our observation that the combined presence of oral *H. pylori* and orthodontic treatment was associated with the highest odds of gastric cancer suggests a possible synergistic association rather than a direct causal relationship, highlighting the potential importance of oral ecological conditions in modulating microbial reservoirs relevant to systemic disease. This interpretation is supported by dental literature demonstrating that orthodontic therapy is associated with significant shifts in oral microbial composition and increased colonization by pathogenic taxa [28,29].

From a broader conceptual perspective, these findings support an ecological model of gastric carcinogenesis in which microbial reservoirs located outside the stomach may be associated with sustained host exposure to carcinogenic bacteria. In this framework, the oral cavity functions not only as a portal of entry to the gastrointestinal tract but also as a dynamic microbial habitat that maintains pathogenic strains over prolonged periods. The role of the oral cavity as a stable reservoir for *H. pylori* remains debated. Some studies have suggested that oral detection may represent transient contamination rather than persistent colonization [18,30]. Nevertheless, the detection of virulence-associated genes and the consistent association with gastric pathology observed in the present study support the possibility that oral niches may harbor biologically relevant bacterial populations. Repeated swallowing of saliva containing *H. pylori*, micro-aspiration events, or episodic gastric recolonization could sustain gastric inflammation even after partial bacterial clearance. This concept aligns with emerging evidence highlighting the role of ectopic microbial colonization in systemic disease and reinforces the importance of considering host–microbiome interactions across anatomical compartments [24,31].

From a translational perspective, these observations emphasize the importance of interdisciplinary collaboration between dental practitioners and gastroenterologists. Orthodontists and dental professionals may represent an important point of early detection and preventive intervention, particularly in populations with a high prevalence of *H. pylori* infection or increased risk of gastric cancer. Enhanced oral hygiene protocols, targeted biofilm control strategies, and increased clinical awareness of gastrointestinal symptoms during orthodontic treatment may represent practical measures to potentially reduce persistent oral microbial reservoirs. Although routine screening for oral *H. pylori* is not currently recommended, our findings raise the possibility that selected high-risk individuals could benefit from integrated oral–gastrointestinal surveillance approaches [30].

This study also contributes to the growing recognition that gastric carcinogenesis is influenced not only by the presence of *H. pylori* but also by the broader ecological context in which the bacterium persists [32]. The oral cavity, as the initial interface between external microbial exposure and the gastrointestinal tract, may play an underappreciated role in shaping microbial translocation, immune activation, and long-term host–microbe interactions [31]. Increasing attention has been directed toward medical and dental interventions capable of altering the oral microbiome and facilitating microbial persistence. Fixed orthodontic appliances represent a particularly relevant ecological environment characterized by increased plaque retention, biofilm maturation, and localized microaerophilic niches that may favor colonization by microorganisms capable of surviving under such conditions [33].

Several limitations of this study should be acknowledged. First, the cross-sectional design precludes temporal assessment of infection dynamics and does not allow determination of whether oral colonization precedes gastric infection or represents secondary colonization from the stomach. Second, molecular detection of *Helicobacter pylori* DNA does not distinguish between viable and non-viable organisms, and culture-based confirmation was not performed. Third, the study population was recruited from a limited clinical setting, and regional differences in *H. pylori* epidemiology may influence the generalizability of these findings to other populations. Additionally, direct molecular comparison between oral and gastric isolates was beyond the scope of the present investigation; strain-level genomic analyses would provide stronger evidence regarding potential transmission pathways between oral and gastric compartments.

Furthermore, the detailed characterization of orthodontic exposure was limited. Information regarding the cumulative duration of orthodontic appliance use before enrollment and detailed bracket design characteristics was not systematically collected. However, all participants with orthodontic treatment used fixed stainless-steel bracket systems with comparable archwire materials, minimizing variability related to appliance material composition. Although the presence of active fixed appliances reflects prolonged biofilm-retentive conditions, the absence of duration data precluded evaluation of potential dose–response relationships between orthodontic treatment and oral *H. pylori* persistence. Future longitudinal studies incorporating precise treatment duration, appliance design characteristics, and detailed biofilm analyses are warranted to clarify whether prolonged orthodontic therapy further increases the likelihood of sustained oral colonization and associated gastric cancer risk.

Finally, molecular detection methods identify bacterial DNA but do not provide information regarding bacterial viability or functional activity. Future studies incorporating culture-based techniques, viability PCR, or metagenomic sequencing approaches would provide additional insight into the biological relevance of oral *H. pylori* detection and its role in the oral–gastric microbial axis.

## 5. Conclusions

This study demonstrates a significant association between oral *Helicobacter pylori* colonization and gastric cancer and identifies fixed orthodontic appliances as a potential ecological factor facilitating oral bacterial persistence. These findings support an expanded associative model of *H. pylori* pathogenesis in which oral microbial reservoirs may be linked to the dynamics of gastric infection. Integrating oral health considerations into strategies for *H. pylori* management may represent a potential direction for interdisciplinary gastric cancer prevention.

## Figures and Tables

**Table 1:** PCR primers used for the detection of *Helicobacter pylori*.

Target Gene	Primer	Primer Sequence (5′ → 3′)	Amplicon Size (bp)
*16S rRNA*	Forward	GCTAAGAGATCAGCCTATGTCC	522
	Reverse	TGGCAATCAGCGTCAGGTAATG	
*ureA*	Forward	AAGCTTTTAGGGGTGTTAGGGGTTT	411
	Reverse	AAGCTTACTTTCTAACACTAACGC	
*glmM* (*ureC*)	Forward	AAGCTTTTAGGGGTGTTAGGGGTTT	294
	Reverse	AAGCTTACTTTCTAACACTAACGC	

**Table 2:** Baseline Characteristics of the Study Population.

Characteristic	Gastric Cancer (*n* = 54)	Precancerous Lesions (*n* = 56)	Non-Atrophic Gastritis (*n* = 52)	Controls (*n* = 50)	*p* Value
Age, years (mean ± SD)	66.4 ± 8.9	61.2 ± 9.7	54.8 ± 10.1	49.6 ± 11.3	<0.001
Male sex, *n* (%)	35 (64.8)	32 (57.1)	28 (53.8)	24 (48.0)	0.18
Smoking, *n* (%)	31 (57.4)	26 (46.4)	21 (40.4)	16 (32.0)	0.03
Active orthodontic treatment, *n* (%)	9 (16.7)	11 (19.6)	8 (15.4)	8 (16.0)	0.91

**Table 3:** Gastric *H. pylori* Positivity Across Diagnostic Groups.

Diagnostic Group	*H. pylori* Positive, *n* (%)	*p* Value
Gastric cancer	41 (75.9)	<0.001
Precancerous lesions	38 (67.9)	0.002
Non-atrophic gastritis	29 (55.8)	0.04
Controls	18 (36.0)	—

**Table 4:** Oral *H. pylori* Detection by Gastric Diagnosis.

Diagnostic Group	Oral *H. pylori* Positive, *n* (%)	*p* Value
Gastric cancer	35 (64.8)	<0.001
Precancerous lesions	30 (53.6)	0.004
Non-atrophic gastritis	21 (40.4)	0.09
Controls	15 (30.0)	—

**Table 5:** Association Between Fixed Orthodontic Treatment and Oral *H. pylori* Colonization.

Orthodontic Status	Oral *H. pylori* Positive, *n* (%)	Oral *H. pylori* Negative, *n* (%)	*p* Value
Active orthodontic treatment	22 (61.1)	14 (38.9)	0.01
No orthodontic treatment	79 (42.0)	109 (58.0)	—

**Table 6:** Oral *H. pylori* Virulence Genes According to Gastric Diagnosis.

Diagnostic Group	*cagA* Positive, *n* (%)	*vacA* Positive, *n* (%)
Gastric cancer	19/35 (54.3)	21/35 (60.0)
Precancerous lesions	12/30 (40.0)	15/30 (50.0)
Non-atrophic gastritis	7/21 (33.3)	9/21 (42.9)
Controls	4/15 (26.7)	5/15 (33.3)

**Table 7:** Multivariable Logistic Regression Analysis for Gastric Cancer.

Variable	Adjusted OR	95% CI	*p* Value
Oral *H. pylori* positivity	3.02	1.51–6.03	0.002
Gastric *H. pylori* positivity	2.41	1.12–5.18	0.02
Active orthodontic treatment	1.89	1.01–3.55	0.046
Oral *H. pylori* + orthodontics	4.18	1.75–9.97	0.001

## Data Availability

The data presented in this study are available from the corresponding author upon reasonable request.
